# Eradication of Small Intestinal Bacterial Overgrowth in Systemic Sclerosis: Current Treatment and Perspectives—A Narrative Review

**DOI:** 10.3390/biomedicines13122932

**Published:** 2025-11-28

**Authors:** Mislav Radić, Andrej Belančić, Marijana Vučković, Almir Fajkić, Marija Rogoznica Pavlović, Josipa Radić

**Affiliations:** 1 Department of Internal Medicine, Division of Rheumatology, Allergology and Clinical Immunology, Center of Excellence for Systemic Sclerosis in Croatia, University Hospital of Split, 21000 Split, Croatia; 2 Internal Medicine Department, School of Medicine, University of Split, 21000 Split, Croatia; 3 Department of Basic and Clinical Pharmacology with Toxicology, Faculty of Medicine, University of Rijeka, Braće Branchetta 20, 51000 Rijeka, Croatia; 4 Department of Internal Medicine, Division of Nephrology and Dialysis, University Hospital of Split, 21000 Split, Croatia; 5 Department of Pathophysiology, Faculty of Medicine, University of Sarajevo, 71000 Sarajevo, Bosnia and Herzegovina; 6 Hospital for Medical Rehabilitation of Heart and Lung Diseases and Rheumatism “Thalassotherapia-Opatija”, 51410 Opatija, Croatia; marija.rogoznica@gmail.com

**Keywords:** systemic sclerosis, small intestinal bacterial overgrowth, SIBO, gut microbiota, gastrointestinal dysmotility, antibiotic therapy, probiotics

## Abstract

Small intestinal bacterial overgrowth (SIBO) is a major yet underrecognized driver of gastrointestinal morbidity in systemic sclerosis (SSc). Disordered motility, fibrosis, and dysbiosis promote microbial stasis, malabsorption, and malnutrition, contributing substantially to impaired quality of life and survival. Diagnostic accuracy remains limited: jejunal aspirate culture is invasive, whereas breath testing offers only moderate sensitivity and specificity. Empirical antibiotic therapy yields transient symptom relief, but recurrence is common, and evidence guiding optimal eradication strategies is sparse. Adjunctive measures, including probiotics, prokinetics, and dietary interventions, remain variably applied, with heterogeneous outcomes across studies. Novel microbiome-targeted, neuromodulatory, and antifibrotic therapies are emerging as promising mechanism-based options. Bearing this in mind, this narrative review aims to consolidate current knowledge on SIBO eradication in SSc. We first outline the pathophysiological rationale and clinical relevance of bacterial overgrowth. We then synthesize available evidence for treatment strategies, appraise barriers to durable remission, and discuss implications for multidisciplinary management. Finally, we highlight emerging approaches, including microbiome-directed therapies, novel prokinetics, and antifibrotic interventions, and define priorities for future clinical research.

## 1. Introduction

Systemic sclerosis (SSc) is a rare connective tissue disease characterized by immune dysregulation, vasculopathy, and progressive fibrosis affecting the skin and internal organs. Clinical outcomes are heterogeneous, dictated largely by the pattern and severity of organ involvement. Despite advances in care, SSc continues to carry the highest morbidity and mortality of all rheumatic diseases [1,2]. Improved survival has shifted the clinical focus toward chronic complications, particularly those involving the gastrointestinal (GI) tract.

GI manifestations are among the most frequent and debilitating features of SSc. Esophageal involvement occurs in up to 90% of patients, while small-bowel disease is reported in 40–70% [3]. GI involvement is not only the leading cause of morbidity but also the third most common cause of mortality in SSc [4]. Hypomotility of the small intestine predisposes to small intestinal bacterial overgrowth (SIBO), a condition that promotes malabsorption and malnutrition and is increasingly recognized as a driver of morbidity and mortality [5]. Patients may experience diarrhea, bloating, weight loss, and micronutrient deficiency, with downstream impacts on function, quality of life, and survival [3,6].

SIBO is now recognized as a central but underappreciated complication of SSc, with prevalence estimates ranging from 30% to 60% depending on cohort and methodology [6]. Fibrotic remodeling of the intestinal wall, impaired autonomic and enteric neural signaling, and vasculopathy combine to disrupt motility and clearance, creating a permissive environment for microbial stasis and dysbiosis. The resulting malabsorption further amplifies nutritional deficits already prevalent in this patient population.

Accurate diagnosis remains a major challenge. The reference standard—microbial culture of jejunal aspirates with >10^3^ colony-forming units per milliliter—is invasive, costly, and poorly suited to routine clinical practice. Breath testing with glucose or lactulose substrates, measuring rises in hydrogen (≥20 parts per million within 90 min) or methane (≥10 parts per million), is more widely applied. These tests are noninvasive and accessible, but diagnostic performance is only moderate, with sensitivity around 60% and specificity near 80% [3,7].

Imperfect concordance between test results, symptom burden, and treatment response further complicates case identification. In practice, clinicians often combine symptom assessment and breath testing with a therapeutic trial of antibiotics for both diagnostic confirmation and initial management [4].

Therapeutic eradication of SIBO can yield rapid gains in nutritional status and symptom control, yet recurrence is common given the persistence of underlying motility impairment. Evidence supporting specific eradication strategies in SSc is limited, and no uniform treatment algorithm exists. Current approaches span antibiotics, probiotics, dietary modification, prokinetics, and supportive care, but data are heterogeneous and practices vary widely [6].

Converging microbiome and metabolomics studies have demonstrated distinct taxonomic and functional dysbioses in SSc patients—changes that correlate with gastrointestinal symptom burden and suggest the gut microbiome as a plausible therapeutic target [8,9,10]. Treatment-focused reviews report consistent symptom and breath-test responses to minimally absorbed antibiotics (notably rifaximin) but also emphasize heterogeneity in study design and the need for antimicrobial stewardship and standardized outcome measures in future trials [11].

This narrative review aims to consolidate current knowledge on SIBO eradication in systemic sclerosis. It included peer-reviewed English-language studies on SIBO eradication in SSc. Studies were prioritized based on methodological rigor, sample size, and clinical relevance, while non-human studies, case reports and unrelated studies were excluded. We first outline the pathophysiological rationale and clinical relevance of bacterial overgrowth. We then synthesize available evidence for treatment strategies, appraise barriers to durable remission, and discuss implications for multidisciplinary management. Finally, we highlight emerging approaches, including microbiome-directed therapies, novel prokinetics, and antifibrotic interventions, and define priorities for future clinical research.

## 2. Pathophysiology and Clinical Relevance of SIBO in Systemic Sclerosis

SIBO in systemic sclerosis (SSc) reflects the intestinal manifestation of the disease’s core pathobiology. It results from the convergence of fibrosis, vascular injury, and immune dysregulation within the gastrointestinal tract. Rather than being a mere complication, it represents a localized expression of systemic processes that progressively disrupt motility, barrier function, and mucosal homeostasis [3,12].

**Figure 1:**
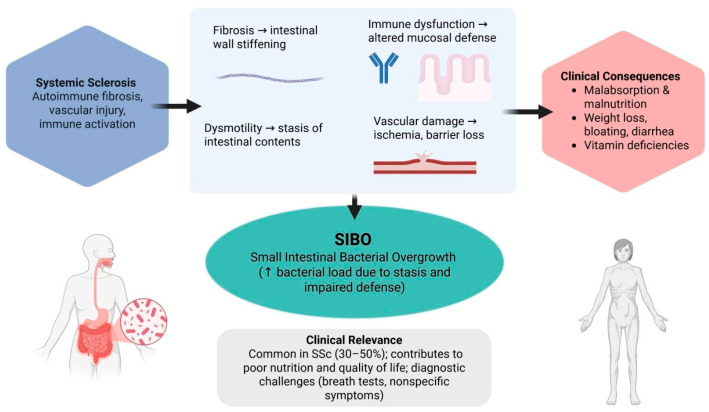
Mechanistic link between systemic sclerosis and small intestinal bacterial overgrowth. Abbreviations: SIBO; Small intestinal bacterial overgrowth SSc; systemic sclerosis.

The interplay between fibrosis, vascular compromise, and immune dysregulation is summarised in Figure 1, highlighting how systemic sclerosis translates into small intestinal bacterial overgrowth and its metabolic consequences.

### 2.1. Fibrotic Foundation: From Structural Rigidity to Functional Stasis

Progressive intestinal fibrosis in SSc is driven by microvascular injury, oxidative stress, and aberrant cytokine signaling mediated by transforming growth factor-β (TGF-β), connective tissue growth factor (CTGF), and platelet-derived growth factor (PDGF) [13,14,15]. Collagen accumulation within the submucosa and muscularis propria stiffens the intestinal wall and diminishes compliance. Smooth muscle cells lose their contractile phenotype, adopting a myofibroblastic state characterized by disorganized extracellular matrix production and reduced peristaltic coordination [14,15]. Persistent activation of TGF-β and CTGF maintains collagen synthesis and decreases elastin content, converting peristalsis into mechanical inertia—the substrate for dysmotility and microbial stasis.

Fibrosis extends into the enteric nervous system, where ischemia and inflammation lead to neuronal loss and reactive gliosis within the myenteric plexus, impairing communication between smooth muscle and intrinsic neural circuits [12]. Disruption of the migrating motor complex (MMC), particularly its phase III clearance waves, results in segmental stasis and retrograde bacterial migration [16,17]. Microbial metabolites such as hydrogen sulfide, predominantly generated by sulfate-reducing species, including *Desulfovibrio* and *Bilophila wadsworthia*, and D-lactate produced by overgrown *Lactobacillus* and *Streptococcus* spp., further amplify oxidative stress and fibroblast activation, perpetuating a self-reinforcing triad of fibrosis, dysmotility, and bacterial proliferation [17,18]. This feedback loop—fibrosis enabling SIBO, and SIBO amplifying fibrosis—typifies the “fibro-inflammatory circuit” that underlies gastrointestinal involvement in SSc.

### 2.2. Dysmotility: The Functional Translation of Structure

Small intestinal dysmotility in SSc stems from fibrotic remodeling, neuropathy, and loss of interstitial cells of Cajal (ICC). Histological studies reveal vascular intimal fibrosis and reduced ICC and neuronal density, impairing slow-wave propagation essential for coordinated peristalsis [19]. The loss of peristaltic gradients prolongs orocecal transit and promotes bacterial reflux. Stasis facilitates bacterial proliferation, which in turn aggravates motility impairment through inflammation and oxidative injury. In advanced disease, quasi-paralytic loops with bile acid stasis and anaerobic overgrowth complete the cycle of structural and functional decline [20].

### 2.3. Immune and Barrier Dysfunction: A Mucosal Amplifier

Immune dysregulation in SSc features endothelial apoptosis, autoreactive B-cell activation, and increased secretion of IL-6 and TGF-β, potent drivers of fibroblast proliferation [21,22,23]. Elevated IL-1, IL-6, IL-17, IFN-α, and TNF-α contribute to endothelial dysfunction and oxidative stress, while loss of tight-junction proteins (occludin, claudin-1) compromises the epithelial barrier and facilitates bacterial translocation [24,25]. SIBO reinforces this inflammatory state via lipopolysaccharide (LPS)–mediated TLR4/MyD88 signaling, stimulating IL-6, IL-17, and TNF-α production and linking dysbiosis directly to fibrotic remodeling [26,27]. Deconjugation of bile acids further disturbs FXR and PPARγ signaling, impairing mucosal repair and perpetuating endotoxemia [28]. Cytokine spillover contributes to anorexia, fatigue, and muscle wasting, establishing the intestine as an immunometabolic amplifier of systemic disease activity [26,27,28].

### 2.4. Vascular Pathophysiology: Ischemia as the Invisible Initiator

Microvascular injury is a hallmark of SSc and a key initiator of intestinal dysfunction. Endothelial damage reduces nitric oxide bioavailability and elevates endothelin-1 levels, producing chronic vasoconstriction and segmental ischemia—an internal counterpart of Raynaud’s phenomenon within the mesenteric circulation [29,30]. Hypoxia stabilizes HIF-1α and HIF-2α, driving maladaptive angiogenesis and VEGF-dependent profibrotic signaling that results in fragile, poorly functional capillaries [31,32]. Ischemic damage to enteric neurons and ICCs exacerbates dysmotility and explains the patchy distribution of SIBO. The vascular origin of these changes also clarifies the incomplete response to prokinetic or antibiotic therapy, which cannot reverse microcirculatory failure [30].

### 2.5. Diagnostic Challenges and Pathophysiological Correlates

Because the clinical and diagnostic aspects are discussed elsewhere, only mechanistic considerations are highlighted here. The sensitivity of glucose or lactulose breath tests is compromised by delayed intestinal transit, while jejunal aspirate cultures, though definitive, are invasive and prone to sampling error [33,34]. Dysmotility itself alters substrate delivery, producing false-negative results in methane-dominant or segmental disease patterns [35]. Hence, diagnostic limitations in SSc largely reflect underlying structural and vascular heterogeneity rather than methodological flaws.

### 2.6. Malnutrition: The Final Common Pathway

SIBO in SSc culminates in malnutrition through bile acid deconjugation, impaired micelle formation, and competitive bacterial uptake of micronutrients [3,36]. Vitamin B12 depletion, fat-soluble vitamin deficiency, and protein-losing enteropathy lead to anemia, coagulopathy, and hypoalbuminemia [36]. Weight loss despite preserved intake indicates both malabsorption and hypercatabolism driven by systemic inflammation. Low BMI and sarcopenia correlate with advanced pulmonary and cardiac fibrosis, confirming that intestinal involvement is not an isolated manifestation but a prognostic indicator of overall disease severity [37].

## 3. Current Treatment Approaches

In SSc, SIBO is a common and clinically significant gastrointestinal complication that causes nutritional deficiencies, bloating, and malabsorption. Beyond its effects on gastrointestinal function, SIBO significantly impacts the quality of life and overall prognosis of SSc patients, underscoring the importance of early detection and targeted treatment [14]. The European Alliance of Associations for Rheumatology (EULAR) identified GI disease in SSc as a high research priority, encouraging studies aimed at improving symptom control and evaluating potential disease-modifying treatments [26].

The aims of SIBO therapy in SSc are symptom relief, microbial eradication, and prevention of recurrence. In this section, several topics will be covered: antibiotics, probiotics, diet, prokinetics, and lifestyle measures, to understand the various approaches to treating SIBO in SSc.

### 3.1. Antibiotic Therapy

When it comes to guidelines, the European Alliance of Associations for Rheumatology (EULAR) recommendations acknowledge that gastrointestinal involvement, including SIBO, is common in systemic sclerosis and suggest sequential or rotating courses of antibiotics as the main therapy, but there is no consensus on the optimal agent, dose, or duration [1]. According to a recent systematic review and meta-analysis, six clinical studies have assessed the efficacy of antibiotics in relieving SIBO-related symptoms, including a total of 196 patients. The trials evaluated the antibiotics rifaximin, norfloxacin, and neomycin, using breath tests or duodenal aspirates for diagnosis and various symptom-based outcome measures. Overall, antibiotic therapy demonstrated a significantly higher clinical response rate (49.5% vs. 13.7%), with a pooled relative risk of improvement of 2.46 (95% CI 1.33–4.55; *p* = 0.004), confirming their effectiveness in managing SIBO symptoms across different patient populations [38].

Wide-spectrum antibiotics targeting Gram-negative aerobes and anaerobes are commonly used for SIBO, often administered for 7–10 days as a single course or in cycles. Recommended options and typical doses include amoxicillin/clavulanate 500/125 mg three times daily, ciprofloxacin 250–500 mg twice daily, doxycycline 100 mg twice daily, metronidazole 250 mg twice or three times daily, neomycin 500 mg twice daily, norfloxacin 400 mg twice daily, rifaximin 550 mg two or three times daily, tetracycline 250–500 mg two to four times daily, and trimethoprim/sulfamethoxazole 160/800 mg twice daily [39].

Rifaximin is the most extensively studied SIBO antibiotic and is preferred for its minimal absorption, fewer systemic side effects, and sustained efficacy, especially in patients with IBS and SIBO; however, it may be costly and less widely available [40]. For intestinal methanogen overgrowth, combining rifaximin with neomycin is often recommended for better results. Rotating antibiotics helps reduce the risk of resistance and is used in recurrent cases [39].

When it comes to SSc-associated SIBO, a study by Parodi et al. on 55 SSc participants and 60 controls reported a 73% eradication rate of SIBO with 1200 mg rifaximin per day for 10 days, assessed one month after treatment [41]. Marie et al. found an eradication rate of 52% after three months of rotating norfloxacin and metronidazole regimens on a sample of 51 SSC participants, further demonstrating that bactericidal therapy, even when used intermittently, is effective for maintenance therapy and long-term remission [42]. A comprehensive overview of studies on antibiotic therapy of SIBO in SSc participants is shown in Table 1.

However, studies to date have been limited by small sample sizes, varying treatment protocols, and frequent recurrences of SIBO, mainly due to gastrointestinal dysmotility that persists in SSc. Frequent use of antibiotics carries greater risks of antibiotic resistance, significant disruption of the gut microbiota, and an increased likelihood of developing infection from Clostridium difficile [43,44]. This long list of adverse effects requires the most careful selection of antibiotics and the most precise directions regarding the duration of treatment, it added.

### 3.2. Probiotics

Although the latest evidence review in the EULAR guidelines did not strengthen findings beyond the 2017 update, the task force—supported by patient representatives—emphasized the need for further research on probiotics and other therapeutic strategies [1].

A meta-analysis of randomized controlled trials involving 176 patients suggests that probiotic supplementation in patients with SSc may relieve certain gastrointestinal symptoms, particularly reflux, gas, and bloating. The interventions used various probiotic strains, including *Lactobacillus paracasei*, *Lactobacillus casei*, *Saccharomyces boulardii*, and combinations with *Bifidobacterium*, with treatment durations ranging from 1 to 8 weeks. Probiotics had a significant positive effect on reflux and bloating, while their impact on constipation, diarrhea, and fecal incontinence was not statistically significant [45].

A randomized study of 40 patients with SSc and SIBO evaluated combination therapy with metronidazole plus *Saccharomyces boulardii*, dividing participants into three treatment arms: metronidazole alone, *Saccharomyces boulardii* alone, and their combination (metronidazole plus *S. boulardii*) [46]. All treatments were administered in repeating cycles over 2 months. The combination of metronidazole and *S. boulardii* demonstrated superior efficacy in eradicating SIBO (55%) compared to either agent alone (33% for *S. boulardii*, 25% for metronidazole). Hydrogen breath test results confirmed a significant post-treatment reduction in hydrogen levels, especially in the combination group. Symptomatically, the *S. boulardii* group improved in gastroesophageal reflux, diarrhea, abdominal pain, and gas/bloating/flatulence; the combination group showed improvements in abdominal pain and gas/bloating/flatulence; and the metronidazole group showed no overall improvement and an increase in abdominal pain and gas/bloating scores. These results suggest that combination antibiotic and probiotic therapy may be more effective for SIBO eradication and symptom relief in systemic sclerosis than either treatment alone. However, the small sample size and large proportion of elimination and follow-up loss should be considered when interpreting these results.

Overall, this evidence suggests that probiotics may be a useful additional treatment for gastrointestinal complications in SSc; however, additional large, high-quality trials are needed to further evaluate their efficacy and safety in this patient population [45].

Due to the rarity of SSc and the underdiagnosis of SIBO, studies remain small and difficult to generalize. Microengineered gut-on-a-chip models offer a potential solution by enabling controlled analysis of human intestinal inflammation, bacterial overgrowth, and barrier dysfunction. These models provide valuable mechanistic insights when patient recruitment and large clinical trials are limited and could indicate new directions for addressing this problem [47].

### 3.3. Dietary Support and Nutritional Management

Malabsorption and unintended weight loss are common complications of SIBO associated with SSc, resulting from impaired nutrient absorption due to bacterial overgrowth [48]. Laboratory investigations in these patients often reveal low levels of hemoglobin, ferritin, total serum protein, phosphorus, calcium, and triglycerides, indicating significant nutritional compromise [12]. In this context, targeted nutritional therapy is crucial not only to address these deficiencies but also to improve quality of life and survival in this population. The specific dietary strategies have been investigated in SIBO participants as follows [48,49,50,51].

An elemental diet made up of free amino acids, simple sugars, and medium-chain triglycerides, which deprives gut microbes of complex substrates while providing essential nutrients in an easily absorbed form, was studied in a recent study involving 30 people with SIBO or intestinal methanogen overgrowth. A two-week exclusive elemental diet was well tolerated and significantly altered the gut microbiome, reducing bacterial taxa such as *Methanobrevibacter smithii* and *Fusobacterium*. It also reduced major gastrointestinal symptoms, including bloating, constipation, and abdominal pain, and normalized lactulose breath tests in 73% of participants [49].

A dietary plan low in fermentable oligosaccharides, disaccharides, monosaccharides, and polyols (known as FODMAPs from Fermentable Oligosaccharides, Disaccharides, Monosaccharides, and Polyols) is frequently recommended for treating irritable bowel syndrome (IBS). It has also gained popularity in managing SIBO [48,50].

Additionally, attention should be given to ensuring that patients consume nutritious foods that are both suitable and recommended, to prevent them from following an inadequate diet that could lead to nutritional deficiencies.

FODMAPs have prebiotic effects and influence the microbiota by stimulating the growth of *Akkermansia muciniphila*, *Bifidobacterium*, and *Faecalibacterium prausnitzii*, as well as promoting the production of short-chain fatty acids [50]. Some authors argue that the negative effects of excessive carbohydrate fermentation outweigh the potential imbalances caused by the diet. Implementing FODMAP approaches is challenging and has limited long-term sustainability. In SSc, a low FODMAP diet did not result in differences in GI symptoms. A study of 66 SSC participants found that greater gastrointestinal symptom severity was associated with reduced microbial diversity and increased abundance of pathobionts such as Klebsiella and Enterococcus. No significant differences in GI symptoms or microbiome diversity were observed between patients following a low FODMAP diet and those on a non-low FODMAP diet, except for higher Enterococcus abundance in the non-low FODMAP group [51].

Nutritional therapy for SIBO in SSc still needs robust scientific evidence since studies are still scarce and of low quality, limiting their clinical applicability. There is a definite lack of randomized trials in SSc-specific populations.

### 3.4. Prokinetic and Motility-Enhancing Therapies

Prokinetic agents are commonly prescribed to alleviate gastrointestinal dysmotility in SSc; however, their long-term medical efficacy is uncertain, and studies are generally limited by small sample sizes and methodological diversity [52].

Several agents have been researched. Domperidone, a dopamine-2 receptor antagonist; low-dose erythromycin; and octreotide, a somatostatin analogue, are the most commonly used prokinetics shown to improve intestinal transit generally or specifically in SSc. Additionally, highly selective 5-hydroxytryptamine receptor agonists, including prucalopride, have proven effective in treating slow-transit idiopathic constipation and are increasingly used for other conditions involving reduced gut motility [4]. Prokinetics may also be used as adjuncts to antibiotic therapy for managing SSc-associated SIBO [52].

The results from the randomized, crossover PROGASS trial involving 40 SSc participants suffering from chronic constipation showed that prucalopride significantly improved the number of complete bowel movements, as well as constipation and reflux scores on validated questionnaires. It also shortened the orocecal transit time. Prucalopride was rated as moderately to extremely effective by 72.4% of participants, while 17.5% discontinued due to adverse events [53].

### 3.5. Lifestyle and Supportive Measures

SIBO lifestyle and supportive measures include eating smaller, more frequent meals, staying upright after eating to encourage motility, and adequate hydration. Physical activity, as tolerated, may help stimulate gut movement [54], and avoiding narcotics and other drugs that reduce motility is recommended [55]. Managing stress is crucial, as there is a correlation between dysfunction of the autonomic nervous system and psychological stress and GI symptoms in SIBO patients [56]. A summary of current therapeutic strategies for managing SIBO in systemic sclerosis is presented in Table 2.

## 4. Challenges in Eradication & Recurrence

### 4.1. High Recurrence Due to Persistent Motility Dysfunction

Early vaso-nervorum impairment of the nerves supplying the GI tract results in impaired function characterized by decreased contractility and advancing fibrosis of the gastrointestinal musculature [57]. The antibiotic failure rate appears high. After a 3-month course of alternating antibiotic therapy using norfloxacin and metronidazole, Marie and colleagues only achieved eradication of SIBO in 31.8% of cases [42]. Additionally, Lauritano et al. reported a relapse rate of 44% for SIBO in patients nine months following successful treatment with rifaximin [58]. Rotating antibiotics does not effectively address the underlying problem because it eliminates both pathogenic and non-pathogenic bacteria, including beneficial probiotic species [46]. The variability of faecal microbiota is greater in individuals with SSc compared to healthy individuals [46].

Quantitative data on SIBO recurrence after eradication therapy in systemic sclerosis (SSc) remain limited, although several studies and a recent meta-analysis provide relevant insights. A systematic review and meta-analysis of 28 studies (*n* = 1112 SSc patients) reported that rifaximin achieved significantly higher eradication rates than rotating antibiotics (77.8 percent [95 percent CI, 64.4–87.9] vs. 44.8 percent [95 percent CI, 31.7–58.4]), but long-term, regimen-specific recurrence rates were not available [8]. The authors also emphasized marked heterogeneity across studies and the limited reliability of breath testing in this population, which must be considered when interpreting these findings.

A prospective study in SSc evaluated a rotating norfloxacin–metronidazole regimen (7 days of norfloxacin 400 mg BID alternated monthly with 7 days of metronidazole 250 mg TID for three months) and reported a 52 percent eradication rate with symptomatic improvement, although no explicit recurrence data were provided beyond immediate follow-up [17]. More robust long-term recurrence data come from general SIBO cohorts. After a single course of rifaximin (1200 mg/day for 7 days), recurrence rates reached 12.6 percent at three months and 27.5 percent at six months on repeat glucose breath testing [5]. These findings are widely cited as representative recurrence patterns, with higher relapse risk observed in older adults, individuals with prior appendectomy, and chronic PPI users [3,58]. However, such estimates likely underestimate recurrence in SSc, given its profound baseline dysmotility and small-bowel involvement [8].

A randomized pilot trial in SSc compared metronidazole, *Saccharomyces boulardii*, and their combination over two months, reporting eradication rates of 25 percent, 33 percent, and 55 percent, respectively, although longer-term recurrence was not assessed [46]. Collectively, current evidence suggests that rifaximin is superior to rotating antibiotics for initial eradication in SSc, but regimen-specific recurrence data remain insufficient.

Recurrence has not been assessed following cyclical gastrointestinal selective antibiotic therapy. In addition to the primary underlying disease, additional risk factors for the recurrence of SIBO have been identified, including prolonged use of proton pump inhibitors (PPIs) (OR 3.5), which affects approximately 90% of SSc patients and may partially account for the lack of SIBO resolution in this population [58].

### 4.2. Antibiotic Resistance, Diagnostic Variability, Patient Heterogeneity

Several experts recommend cyclical antibiotic regimens for patients with recurrent, culture-confirmed SIBO, as evidenced by endoscopically obtained duodenal aspirates. This treatment plan usually calls for giving antibiotics for 10 to 14 days each month, with the recommended drugs being switched out every so often to keep antimicrobial resistance from happening [59,60].

Clinical symptom questionnaires serve primarily for the detection or investigation of SIBO. While culture and bacterial count from small bowel fluid aspiration are considered the gold standard, they are associated with a higher incidence of false positives due to contamination or technical challenges [6]. Therefore, diagnosis of SIBO is primarily made using a noninvasive breath test that measures exhaled hydrogen induced by intestinal bacteria fermentation of carbohydrates, such as glucose, lactulose, or d-xylose [6]. Lactulose, a non-absorbable synthetic disaccharide with prebiotic effects, is generally well-tolerated; however, intestinal discomfort and transient diarrhoea may occasionally occur during testing [61]. No statistically significant association was found in studies utilizing lactose as the substrate; however, a significant association was observed in those employing glucose, indicating potential differences in substrate effects [6]. False positives are infrequent but may arise due to contamination with pharyngeal bacteria, a fiber-rich diet, or changes in ventilation [62,63]. False negatives may arise when bacteria responsible for degrading indigestible carbohydrates are absent, which can result from recent antibiotic use, laxative administration, enemas, or acute diarrhoea during the testing period [64]. A North American Consensus Group did not advise discontinuing PPIs prior to breath testing [65]. Diagnostic ambiguity in SSc-related SIBO often mirrors the underlying pathophysiology -segmental fibrosis, vascular ischemia, and disrupted motility create spatially heterogeneous niches where overgrowth may wax and wane, leading to inconsistent test results and fluctuating clinical presentations.

Recent diagnostic developments aim to address the well-recognized limitations of conventional breath testing in systemic sclerosis, where severe dysmotility often leads to false-positive or false-negative results. Molecular approaches, including 16S rRNA sequencing and shotgun metagenomics of duodenal aspirates, now allow direct characterization of the small-bowel microbiome, consistently demonstrating reduced microbial diversity and an overrepresentation of Proteobacteria such as *Escherichia coli* and *Klebsiella* spp. These methods also identify metabolic pathways linked to hydrogen and hydrogen-sulfide production, offering more precise phenotyping of SIBO in SSc, although they currently remain research tools rather than routine diagnostics [66,67].

Novel device-based techniques, most notably intraluminal gas-sensing capsules, permit real-time measurement of hydrogen, methane, carbon dioxide, and oxygen during small-bowel transit. Early studies report high concordance with duodenal aspirate cultures and superior accuracy to traditional breath tests, suggesting particular value in SSc, where delayed transit complicates interpretation of breath profiles [68].

Biomarker-based strategies remain limited, but some evidence suggests that fecal calprotectin may be elevated in patients with significant mucosal inflammation secondary to bacterial overgrowth, including in systemic sclerosis. However, performance is inconsistent, and it is not recommended as a stand-alone diagnostic marker [5]. Metabolomic indicators, including fecal lactate patterns and D/L-lactate ratios, may help identify patients at risk for complications such as D-lactic acidosis, particularly in those with profound dysmotility, but these signatures still require further validation before integration into routine SSc care [69].

Reports indicate that patients with the diffuse subtype exhibit a greater prevalence of gastrointestinal symptoms, while those with anticentromere antibodies demonstrate a reduced risk [70]. Conversely, the lack of anti-topoisomerase-I antibodies has been noted in patients without SIBO [57]. These findings suggest that prolonged disease duration may serve as a predisposing factor for SIBO, whereas the absence of anti-topoisomerase-I antibodies may confer a protective effect in SSc patients [57]. Nevertheless, these findings necessitate validation in more extensive, prospective studies.

### 4.3. Clinical and Research Barriers

Given the limited knowledge about gastrointestinal manifestations and the robust support from patient representatives, the EULAR task group highly prioritized gastrointestinal illness research in SSc [1]. Since there are no randomized controlled trials, the interventional studies on treating SIBO in individuals with SSc are generally of low quality. The Cochrane Risk of Bias states that, as there is no blinding, randomization, or allocation concealment, there is typically a significant risk of bias [71]. Additionally, there were cases of selective and insufficient data reporting. Future research should encompass the identification of treatments and interventions for improved symptom management, as well as the evaluation of the impact of disease-modifying therapy on the natural progression of gastrointestinal symptoms in SSc. There is a need for further research, especially research that addresses the specific effects of probiotics.

## 5. Clinical Practice Implications

### 5.1. Practical Management Approach

The EULAR recommendations for the treatment of systemic sclerosis, update 2025, upheld the suggestion for the utilization of rotating antibiotics in the therapy of SIBO, grounded in interventional research [1]. The therapeutic strategy is the oral administration of amoxicillin during the first month (500 mg 3×/24 h), ciprofloxacin during the second month (500 mg 2×/24 h), and metronidazole during the third month (500 mg 3×/24 h) [12]. One interventional study evaluated a motility agent for the treatment of SIBO in SSc [72], whereas the other four uncontrolled studies examined various antibiotic regimens [41,42,57,73]. Patients treated with octreotide [72] or ciprofloxacin [63] had 100% eradication rates; those treated with rifaximin [41] had 73.3%; those treated with intermittent rotating norfloxacin and metronidazole [42] had 52.4%; and those treated with amoxicillin, ciprofloxacin, and metronidazole for one month each had 43% [57]. A meta-analysis was not possible due to the heterogeneity of the trials and the absence of treatment control arms [6]. Another study by Shah SC et al. [59] examined the eradication of SIBO using metronidazole and rifaximin, achieving rates of 51.2% and 49.5%, respectively.

Quantitative data concerning the recurrence of SIBO in SSc subsequent to various eradication protocols are scarce. While medications may eliminate SIBO in many SSc patients, a significant proportion continue to exhibit SIBO symptoms at the 11-month follow-up [42].

The effectiveness appears to be enhanced with the sequence of antibiotics administered after the use of *Lactobacillus* as a bacterial probiotic [74]. A study by García-Collinot, G. et al. demonstrated the partial efficacy of metronidazole treatment in SIBO; however, the use of *S. boulardii*, either as monotherapy or in combination, enhances gastrointestinal outcomes in SSc [46].

PPIs exhibit increased duodenal bacterial overgrowth, likely resulting from a decrease in gastric acid production [75]. Reducing PPIs in SSc when feasible may aid in preventing SIBO, acknowledging that this is frequently unachievable due to esophageal peristalsis, esophageal sphincter atony, and ongoing gastroesophageal reflux [76].

### 5.2. Multidisciplinary Care Model

Managing gastrointestinal problems related to SSc requires a team approach, involving gastroenterologists, rheumatologists, and nutritionists [36]. The main treatment for SIBO in SSc should include changes to diet and lifestyle, along with medications [4]. Nutritional interventions are crucial for reducing gastrointestinal symptoms, preventing malnutrition and its related health issues, and ultimately improving the patient’s quality of life (QoL) [77].

### 5.3. Emphasis on Quality of Life and Individualized Therapy

Gastrointestinal symptoms significantly affect the health-related QoL in SSc patients [78]. Both the upper and lower parts of the gastrointestinal tract can be affected by SSc, potentially leading to significant negative impacts on QoL [78,79]. SIBO can further worsen a patient’s QoL and may cause serious complications, such as malnutrition and a higher risk of infections [57]. The main goal of treating SIBO is to address any underlying disease or structural problem that contributes to its development [4]. Conversely, achieving this within the framework of SSc is, for the most part, not feasible. Therapeutic approaches, contingent upon the specific clinical manifestations and overall patient health, may encompass antibiotic and prokinetic treatments, probiotic supplementation, and, in select instances, intravenous immunoglobulin (IVIG) administration. Furthermore, the identification and rectification of nutritional deficiencies through suitable interventions, including nutritional support and/or supplementation with fat-soluble vitamins, vitamin B12, iron, and folate, is of paramount importance [4].

The practical flowchart of managing SIBO is presented below as follows:

1. **Symptoms suggest SIBO** → Bloating, diarrhea, pain, distension
2. **Diagnose or Empirically Treat** → Breath test if available; otherwise treat based on symptoms
3. **First-Line Treatment** → Rotating antibiotics (amoxicillin → ciprofloxacin → metronidazole); alternatives: rifaximin, norfloxacin + metronidazole, ciprofloxacin, octreotide
4. **Adjunctive Measures** → Probiotics (*S. boulardii*, *Lactobacillus*); reduce PPIs; consider prokinetics
5. **Nutrition** → Check deficiencies (A, D, E, K, B12, iron, folate) and provide supplementation
6. **Reassess** → If persistent, change regimen or repeat rotation
7. **Multidisciplinary Care** → Gastroenterologist + Rheumatologist + Nutritionist

## 6. Emerging and Future Perspectives

Future progress in the management of GI manifestations in SSc will likely come from three areas: microbiome-directed interventions, selective prokinetics, and antifibrotic therapies. Current antibiotic-based regimens achieve inconsistent and often short-lived effects, indicating the need for treatments that modify underlying mechanisms rather than symptoms alone [6].

Early and characteristic dysbiosis in SSc, together with its links to mucosal immune function and motility, suggests a possible pathogenic contribution rather than a secondary phenomenon [80]. Pilot FMT studies have demonstrated biological activity, including transcriptomic changes in the duodenum [81], although the clinical benefit remains variable. The first randomized trial with anaerobically cultivated microbiota (ACHIM) confirmed safety without clinical superiority to placebo [82]. These findings emphasize the importance of standardized microbial products, improved donor–recipient matching, and validated clinical endpoints. Future work should define SSc-specific microbial signatures, develop targeted microbial consortia, and individualize nutritional strategies using microbial and metabolomic profiling.

GI dysmotility remains a major therapeutic gap. Standard prokinetics provide modest and short-lived benefit and carry safety concerns [83]. More selective 5-HT4 agonists, such as prucalopride, have improved symptoms in small SSc cohorts [53]. Relamorelin, a ghrelin receptor agonist, accelerated gastric emptying in phase 2 trials of diabetic gastroparesis, offering a plausible translational option for SSc [84]. Intranasal metoclopramide demonstrated faster onset and better symptom control compared with oral formulations in a recent phase 3 study [85], indicating that targeted receptor modulation and alternative delivery routes should be evaluated in SSc.

Limited observational data suggest that IVIG may help refractory dysmotility and intestinal pseudo-obstruction, particularly when autoimmune mechanisms of enteric neuropathy or myopathy are suspected [86]. Interventional approaches such as gastric peroral endoscopic pyloromyotomy (G-POEM) and sacral nerve stimulation (SNS) have shown feasibility in severe gastroparesis and anorectal dysfunction and merit assessment in dedicated SSc trials [87].

Progressive intestinal fibrosis drives irreversible motility loss and malnutrition. Antifibrotic therapies used in other SSc manifestations represent a logical direction. Nintedanib slows fibrotic progression in interstitial lung disease [88], while belumosudil modulates ROCK2-dependent signaling pathways involved in inflammation and fibrosis [89]. Inhibition of LOXL2, a key enzyme in collagen cross-linking, is also under investigation [90]. Combining antifibrotic agents with neuromodulatory or prokinetic strategies may address both structural and functional pathology.

Given the heterogeneity of SSc, no single therapeutic model will likely fit all patients. A “treat-to-target” approach for GI involvement would require validated motility testing, biomarker development, microbiome profiling, robust patient-reported outcomes, and integration with digital symptom tracking. Large registries linked to molecular datasets will be essential for identifying predictors of treatment response and assessing real-world effectiveness [83].

Microbiome-based therapies remain promising but largely experimental. Early FMT and ACHIM trials demonstrate biological activity without consistent symptomatic improvement, highlighting the limitations of nonspecific microbial transfer [81,82]. Future approaches will likely depend on engineered microbial strains and rationally assembled consortia with defined metabolic and immunomodulatory functions, as demonstrated in engineered-microbiota studies [82]. In parallel, non-microbiome strategies, including next-generation prokinetics, IVIG, G-POEM, SNS, and emerging antifibrotic agents, are expected to contribute meaningfully when integrated into phenotype-guided treatment algorithms.

Preclinical and clinical research on SIBO in patients with SSc is still mainly neglected. Although therapy has advanced, there are still significant gaps in its management. In order to enhance long-term outcomes for patients with SIBO, optimize symptom control, and refine treatment options, larger, well-designed studies are desperately needed.

## 7. Conclusions

SIBO is a central gastrointestinal complication of SSc, arising from fibrosis, dysmotility, vascular injury, and immune dysregulation. Current therapies, including antibiotics, probiotics, dietary modification, and prokinetics, provide symptomatic relief and transient microbial eradication, yet recurrence is frequent due to persistent motility impairment, structural intestinal changes, proton pump inhibitor use, and antibiotic resistance. SIBO contributes to malnutrition, micronutrient deficiencies, and systemic inflammation, linking intestinal involvement to overall disease severity and prognosis. Emerging interventions such as microbiome-directed therapies, selective prokinetics, intravenous immunoglobulin, antifibrotic agents, as well as interventional techniques such as G-POEM and sacral nerve stimulation, offer promise for mechanism-based disease modification. Future management should adopt a precision-guided, multidisciplinary approach, integrating validated motility assessments, microbial and molecular biomarkers, patient-reported outcomes, and digital symptom tracking. Large registries and adaptive clinical trials will be essential to identify predictive markers and optimize real-world effectiveness, ultimately improving quality of life and long-term outcomes in SSc-associated SIBO.

## Figures and Tables

**Table 1:** Comparative summary of key clinical studies on antibiotic therapy for small intestinal bacterial overgrowth in systemic sclerosis.

Study	Population	Intervention(s)	Outcomes	Limitations
Parodi et al., 2008 [41]	55 SSc patients (50F/5M; mean age 59 ± 11 years); 18 diffuse, 37 limited SSc; 60 controls. SIBO diagnosed via lactulose breath test (LBT).	Rifaximin 400 mg TID (1200 mg/day) for 10 days. Reassessment after 1 month via LBT and symptom score.	73.3% eradication (22/30 SIBO+); >50% GSS reduction (8 → 2, *p* < 0.05); Improved diarrhea, bloating, pain.	Small cohort; 1-month follow-up; open-label design; no control group; all patients on PPIs.
Marie et al., 2009 [42]	51 SSc patients (41F/10M; median age 54 [23,24,25,26,27,28,29,30,31,32,33,34,35,36,37,38,39,40,41,42,43,44,45,46,47,48,49,50,51,52,53,54,55,56,57,58,59,60,61,62,63,64,65,66,67,68,69,70,71,72,73,74,75,76,77,78,79,80,81,82]); median disease duration 4 years; 25 diffuse/26 limited SSc. 43% SIBO+ via glucose breath test.	Rotating antibiotics: Metronidazole 250 mg TID + Norfloxacin 400 mg BID Administered 7 days/month for 3 months.	31.8% eradication at 3 months; 52.4% at 6 months; GSS improved significantly (8.5 → 1.5, *p* < 0.001).	Open-label; small sample; variable follow-up; no control group; limited long-term relapse data.

Abbreviations: SSc—Systemic sclerosis, SIBO—Small intestinal bacterial overgrowth, LBT—Lactulose breath test, GSS—Global Symptom Score, PPI—Proton pump inhibitor, TID—Three times daily, BID—Twice daily.

**Table 2:** Summary of Treatment Approaches for SIBO in Systemic Sclerosis.

Approach	Main Agents/Interventions	Evidence Summary	Benefits	Limitations
Antibiotics	Rifaximin, Norfloxacin, Metronidazole, Neomycin	Meta-analysis (*n* = 196): 49.5% response vs. 13.7%. Rifaximin 73% eradication; rotating regimens effective.	Effective, well-studied, short courses	Recurrence, resistance, microbiota disruption, C. difficile
Probiotics	*L. paracasei*, *L. casei*, *S. boulardii*, *Bifidobacterium*	Meta-analysis (*n* = 176): improved bloating/reflux. RCT: *S. boulardii* + metronidazole 55% eradication.	Adjunctive benefit, symptom relief	Small trials, variable efficacy
Dietary/Nutritional	Elemental diet; low FODMAP	Elemental diet: 73% normalized breath test; FODMAP—microbial changes, no symptom difference.	Nutritional support, symptom relief	Limited SSc-specific data
Prokinetics	Domperidone, Erythromycin, Octreotide, Prucalopride	PROGASS trial (*n* = 40): improved bowel movements, reflux scores.	Improves motility, adjunctive effect	Adverse events, limited data
Lifestyle	Small meals, hydration, upright posture, stress control	Supports motility and symptom relief.	Safe, supportive	Evidence limited

## Data Availability

No new data were created or analyzed in this study.
